# Yiqi Huayu decoction alleviates bleomycin-induced pulmonary fibrosis in rats by inhibiting senescence

**DOI:** 10.3389/fphar.2022.1033919

**Published:** 2022-10-28

**Authors:** Biao Zuo, Ling Zuo, Xu-Qin Du, Su Yuan, Chen Xuan, Yu-Di Zhang, Zhi-Wei Chen, Wen-Fu Cao

**Affiliations:** ^1^ College of Traditional Chinese Medicine, Chongqing Medical University, Chongqing, China; ^2^ Chongqing Key Laboratory of Traditional Chinese Medicine for Prevention and Cure of Metabolic Diseases, Chongqing, China

**Keywords:** Yiqi Huayu decoction, pulmonary fibrosis, network pharmacology, cellular senescence, senescence-associated secretory phenotypes

## Abstract

**Overview:** In treating pulmonary fibrosis (PF), traditional Chinese medicine (TCM) has received much attention, but its mechanism is unclear. The pharmacological mechanisms of TCM can be explored through network pharmacology. However, due to its virtual screening properties, it still needs to be verified by *in vitro* or *in vivo* experiments. Therefore, we investigated the anti-PF mechanism of Yiqi Huayu Decoction (YHD) by combining network pharmacology with *in vivo* experiments.

**Methods:** Firstly, we used classical bleomycin (BLM)-induced rat model of PF and administrated fibrotic rats with YHD (low-, medium-, and high-dose). We comprehensively assessed the treatment effect of YHD according to body weight, lung coefficient, lung function, and histopathologic examination. Second, we predict the potential targets by ultra-high-performance liquid chromatography-tandem mass spectrometry (UHPLC-MS/MS) combined with network pharmacology. In brief, we obtained the chemical ingredients of YHD based on the UHPLC-MS/MS and TCMSP database. We collected drug targets from TCMSP, HERB, and Swiss target prediction databases based on active ingredients. Disease targets were acquired from drug libraries, Genecards, HERB, and TTD databases. The intersecting targets of drugs and disease were screened out. The STRING database can obtain protein-protein interaction (PPI) networks and hub target proteins. Molecular Complex Detection (MCODE) clustering analysis combined with enrichment analysis can explore the possible biological mechanisms of YHD. Enrichment analyses were conducted through the R package and the David database, including the Kyoto Encyclopedia of Genes and Genomes (KEGG), Gene Ontology (GO), and Reactome. Then, we further validated the target genes and target proteins predicted by network pharmacology. Protein and gene expression detection by immunohistochemistry, Western blot (WB), and real-time quantitative PCR (rt-qPCR).

**Results:** The results showed that high-dose YHD effectively attenuated BLM-induced lung injury and fibrosis in rats, as evidenced by improved lung function, relief of inflammatory response, and reduced collagen deposition. We screened nine core targets and cellular senescence pathways by UHPLC-MS/MS analysis and network pharmacology. We subsequently validated key targets of cellular senescence signaling pathways. WB and rt-qPCR indicated that high-dose YHD decreased protein and gene expression of senescence-related markers, including p53 (TP53), p21 (CDKN1A), and p16 (CDKN2A). Increased reactive oxygen species (ROS) are upstream triggers of the senescence program. The senescence-associated secretory phenotypes (SASPs), containing interleukin 6 (IL-6), tumor necrosis factor-alpha (TNF-α), and transforming growth factor-β1 (TGF-β1), can further exacerbate the progression of senescence. High-dose YHD inhibited ROS production in lung tissue and consistently reduced the SASPs expression in serum.

**Conclusion:** Our study suggests that YHD improves lung pathological injury and lung function in PF rats. This protective effect may be related to the ability of YHD to inhibit cellular senescence.

## 1 Introduction

Pulmonary fibrosis (PF) is a chronic, progressive, and irreversible fibrotic lung disease, and its prominent feature is matrix stiffening (Moss et al., 2022). Symptoms of PF are mainly characterized by worsening dyspnea and ventilatory and ventilatory dysfunction (Martinez et al., 2017). Patients with PF have an inferior prognosis, with hypoxemia and respiratory failure leading to death (Moss et al., 2022). With a median survival of only 2.5–3.5 years, PF’s terrible prognosis rivals some of the worst malignancies. With a high mortality rate and incurable properties, PF has received more attention, especially after the peak of the Coronavirus disease outbreak in 2019 (COVID-19) (Gentile et al., 2020; Spagnolo et al., 2020; Aul et al., 2021; Tanni et al., 2021). However, there are currently no radical drugs in clinical practice to cope with this devastating lung disease (Spagnolo et al., 2021). Therefore, the development of effective treatment for PF is urged.

PF is an aging-related disease, and cellular senescence is crucial in the aging phenotype (Schafer et al., 2017). The accelerated senescence of alveolar epithelial cells is increasingly recognized as a primary cause of epithelial dysfunction and PF pathogenesis (Yao et al., 2021). Senescent alveolar epithelial cells not only lose their ability to regenerate and repair, but also exert deleterious effects on neighboring cells by secreting various pro-inflammatory cytokines, profibrotic factors, and growth factors (Kadota et al., 2018). Those secretions are defined as senescence-associated secretory phenotypes (SASPs). Recently, senotherapeutics and senolytics have become emerging hotspots (Justice et al., 2019; Merkt et al., 2020; Wissler Gerdes et al., 2021).

Because TCM can improve patient quality of life and survival rate, it has enormous potential in treating PF(Li and Kan, 2017). Yiqi Huayu decoction (YHD), a modified traditional Chinese prescription, has been applied to the clinical treatment of PF(Zhang et al., 2018). YHD consists of two botanical drugs: *Astragalus* mongholicus Bunge and Salvia miltiorrhiza Bunge. This prescription can improve PF by tonifying the lung, benefiting Qi, activating blood circulation, and removing blood stasis. Increasing evidence suggests that YHD can potentially prevent or treat various fibrotic diseases by suppressing inflammatory responses, inhibiting myofibroblast activation, and promoting collagen degradation (Qin et al., 2018; Han et al., 2021). The clinical application of YHD in PF has been validated, but its mechanism is unclear because its components and targets are complex. Therefore, it is valuable to further investigate the mechanism of YHD in treating PF.

**FIGURE 1:**
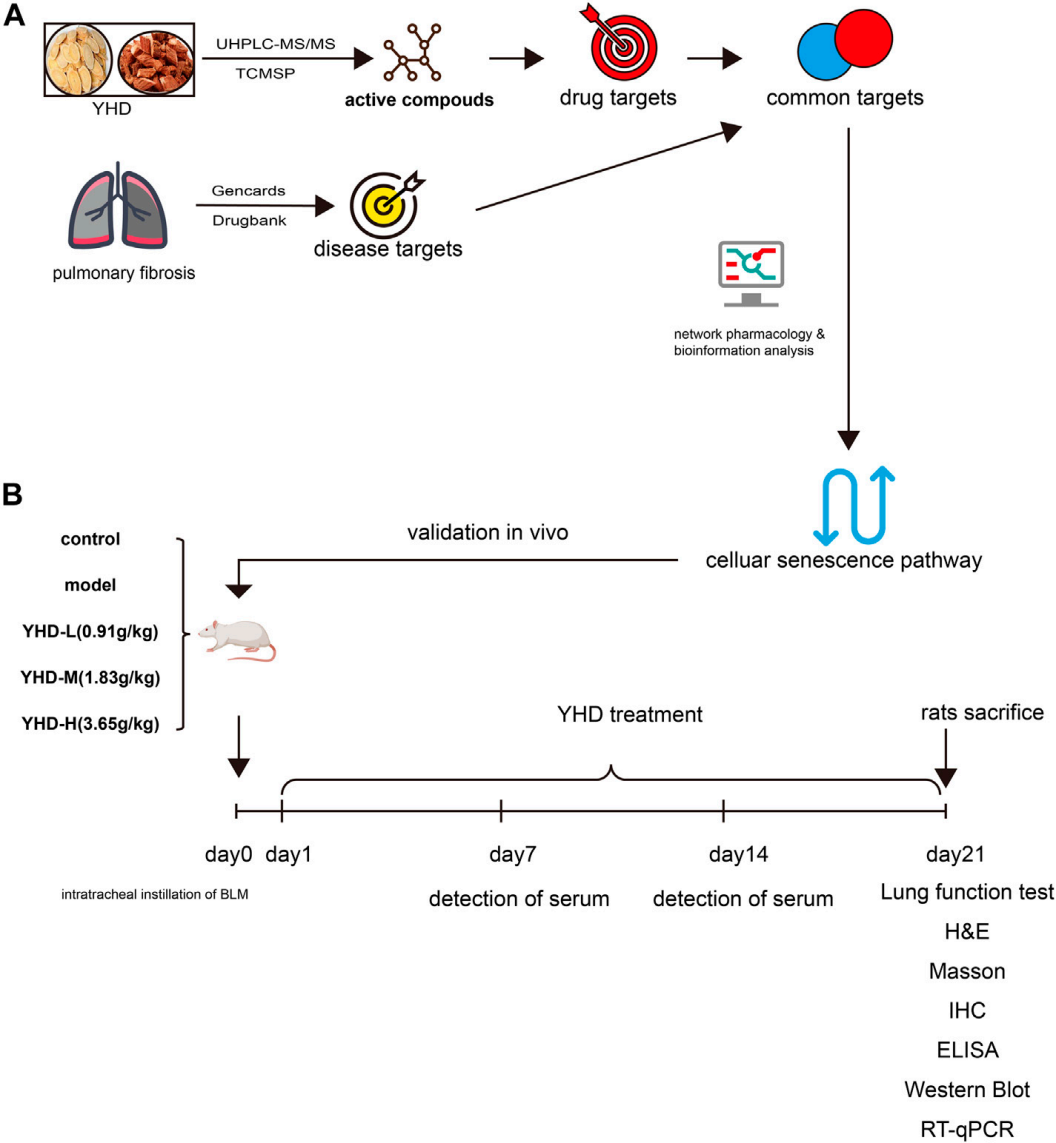
Experimental flow diagram. **(A)**Network pharmacology **(B)** Animal experiment. Pulmonary fibrosis was induced in rats by tracheal drops of bleomycin at day 0. From day 1 to day 21, rats were given YHD by gavage once a day, and orbital blood was collected from rats once a week. Each group of rats ended up with at least 6 biological replicates.

In our study, we used a classical bleomycin-induced rat model of PF and administered different doses of YHD. The therapeutic effect of YHD was evaluated comprehensively by body weight, lung coefficient, survival rate, lung function, and pathological sections of rats. Network pharmacology is an effective tool for predicting complex pharmacological mechanisms and has been widely used by TCM researchers. Through component identification (UHPLC-MS/MS analysis) and network pharmacology, we screened out key targets and signaling pathways to reveal the mechanism of YHD in treating PF. We then further validated key markers on cellular senescence signaling pathways with different approaches. An overview of our study is shown in Figure 1.

## 2 Materials and methods

### 2.1 Drugs and reagents

YHD granules are provided by Yifang Pharmaceutical (Guangdong, China) to ensure accurate dosage. Each herb underwent a series of processes, including decocting, extracting, concentrating, drying, and finally preparing into granules. These granules were identified by professor Wen-fu Cao of Chongqing Medical University. The granules sample are saved in Chongqing Key Laboratory of Traditional Chinese Medicine for Prevention and Cure of Metabolic Diseases. Detection kits for hydroxyproline are available from Nanjing Jiancheng Bioengineering Institute (Nanjing, China). Detection kits for reactive oxygen species (ROS) are available from Nanjing Fengfeng Biomedical Technology Co. (Nanjing, China). The TGF-β1, IL-6, and TNF-α detection kits were provided by Jiubang Biotechnology (Fujian, China). All reagents for rt-qPCR were from Takara Bio (Kusatsu, Japan). All primers for qPCR were synthesized by Tsingke Biology Technology (Beijing, China). Acetonitrile, methanol, and formic acid (LC-MS grade) were purchased from CNW Technologies (Dusseldorf, Germany). Antibodies against GAPDH (ab181602), p16 (ab51243), and P21 (ab109199) were offered by Abcam (Cambridge, United Kingdom). Antibodies against P53 (sc-99) were purchased from Santa Cruz. Antibodies against Collagen I (GB11022-3), Collagen Ⅲ(GB111629), and *α*-SMA (GB111364) were acquired from Servicebio (Wuhan, China).

### 2.2 Preparation of Yiqi Huayu decoction

YHD consists of two botanical drugs: *Astragalus* mongholicus Bunge (AM) and Salvia miltiorrhiza Bunge (SM). Table 1 shows detailed YHD information and composition ratios. All original medicinal materials are made into granules according to the procedures of the Chinese Pharmacopoeia. Specifically, the original herbs were soaked in 7 times the volume of purified water for 30 min, brought to a boil over high heat, and continued to decoct for 60 min. After filtering off the liquid, water was added again, brought to a boil over high heat, and the decoction was continued for 40 min. Finally, the liquid obtained from the two decoctions was mixed, dried, concentrated, and packaged into granules. 60 g of raw astragalus was concentrated into 12 g of granules, and 30 g of salvia was concentrated into 5.4 g of granules. The YHD oral liquids were made by mixing granules of single botanical drugs with double-distilled water and dissolving them. The daily dose of YHD granules in adults is 0.29 g/kg. Rats’ daily dose was calculated as 1.827 g/kg using the conversion ratio of surface area between rats and humans (6.3). This dose was used as the medium dose. The low dose of YHD was 0.9135 g/kg, whereas the high dose was 3.654 g/kg.

**TABLE 1:** Compositions of YHD.

Scientific name	Botanical dosage (g)	Corresponding dosage of granules (g)	Occupied percent (%)
*Astragalus* mongholicus Bunge [Fabaceae; Astragali radix]	60	12	68.97
Salvia miltiorrhiza Bunge [Lamiaceae; Salviae miltiorrhizae radix et rhizoma]	30	5.4	31.03

### 2.3 Ultra-high-performance liquid chromatography tandem mass spectrometry

100 mg YHD sample dissolved in 500ul extraction solution (Methanol: water = 4:1, the internal standard concentration is 10 ug/mL). Vortex for 30 s, sonicate at 45 Hz for 4 min, and sonicate in an ice-water bath for 1 h; After standing at - 40°C for 1 h, the sample was centrifuged at 4°C, 12,000 rpm (centrifugal force 13,800 (×g), radius 8.6 cm) for 15 min; The supernatant was filtered through a 0.22 um microporous membrane. 5ul filtered supernatant was detected for UHPLC-MS/MS analysis.

### 2.4 Animals

Thirty adult male Sprague-Dawley rats (weighing 200–220 g) were used as research subjects. All animals were supplied by the Laboratory Animal Center of Chongqing Medical University and kept in a specific pathogen-free room at the center. The laboratory was maintained at 22.9 (°C) with a relative humidity of 46.4% and a 12-h dark photoperiod. All rats were fed standard chow and water for 7 days in the laboratory before the experiment.

### 2.5 Model preparation and administration

After 7 days of adoption, all rats were randomly divided into five groups (n = 6 in each group) as follows: control group, model group, YHD at low-, medium-, and high-dose group (0.9135,1.827, 3.654 g/kg/day). On day 0, rats in the control group were treated with an equal volume of saline, and the other groups’ rats were intratracheally injected with BLM dissolved in saline. Briefly, rats were anesthetized (2% sodium pentobarbital) and instilled with BLM solution (5 mg/kg) by the intratracheal route. From day 1 to day 21, rats in the control and model groups were treated with saline, while rats in the low-, medium-, and high-dose YHD groups were treated with the corresponding doses of YHD. All rats were sacrificed on day 21st. The lower 1/3 of the left lung was fixed with 4% paraformaldehyde and sectioned for H&E, MASSON, and immunohistochemical staining. The remaining lung tissues were stored at -80°C for future analyses.

### 2.6 Lung function test

On day 21st, rat lung function was measured using the FinePointe non-invasive testing system (FinePointe™ NAM, Data Sciences International). Specifically, the rat is secured in a closed box with an airflow monitoring apparatus attached to one end of the box. Different respiratory parameters such as breathing frequency (F), minute volume (MV), tidal ventilation volume (TV), specific airway conductance (sGaw), functional residual capacity (FRC), and specific airway resistance (sRaw) were derived from the airflow. The average values calculated by the system are counted as raw data.

### 2.7 Hydroxyproline and reactive oxygen species

Hydroxyproline (HYP) contents in lung tissues were measured using the Hydroxyproline Assay Kit (Nanjing Jiancheng Corp. Nanjing, China) according to the manufacturer’s instructions. In brief, 50 mg of lung tissue was mixed with 1 ml of hydrolase and placed in a boiling water bath for 20 min. The supernatant was centrifuged at 3,500 rpm/min for 10 min, and the absorbance was measured at 550 nm. The detection of reactive oxygen species (ROS) in lung tissue is according to the kit instructions. Specifically, 50 mg of lung tissue was homogenized in 1 ml of homogenizing Buffer, and the supernatant was harvested by centrifugation. We sequentially added 200 ul of supernatant and 2 μl of liquid containing the fluorescent probe to the 96-well plate. Fluorescence intensity was measured at excitation wavelength 510 nm and emission wavelength 610 nm. After measuring the protein concentration of the supernatant, the fluorescence intensity/protein concentration indicates the intensity of tissue reactive oxygen species.

### 2.8 Histological analysis and immunohistochemistry

The left lung tissues were fixed in 4% paraformaldehyde for 24 h, embedded in paraffin. According to the manufacturer’s instructions, histological sections were used for hematoxylin-eosin (H&E), Masson, and immunohistochemical (IHC) experiments. Alveolitis was assessed with H&E-stained sections, and fibrosis was assessed with Masson’s trichrome-stained sections. The inflammation and fibrosis scores were assessed quantitatively based on previous literature (Szapiel et al., 1979). All the sections were analyzed by microscopy (BX53, Olympus Corporation, Japan).

#### 2.8.1 Hematoxylin-eosin Staining

Paraffin sections were deparaffinized, stained with hematoxylin staining solution for 5 min, rinsed with distilled water, dehydrated with graded alcohol, and then stained with eosin staining solution for 5 min, and dehydrated and sealed. Sections were rinsed with 1% glacial acetic acid for differentiation, dehydrated with absolute ethanol, and fixed.

#### 2.8.2 Masson staining

Sections were immersed in Masson A solution overnight and washed with water. And the sections were filled into a dye solution mixed with Masson B solution and Masson C solution in equal proportions, soaked for 1 min, washed with water, differentiated with 1% hydrochloric acid alcohol, and washed with water. Next, the sections were plunged into Masson D solution for 6 min, rinsed with water, and plunged into Masson E solution for 1 min. After a slight drain, the sections were directly stained with Masson F solution for 2–30 s. Sections were rinsed with 1% glacial acetic acid for differentiation, dehydrated with absolute ethanol, and fixed.

#### 2.8.3 Immunohistochemistry

We incubated the sections at 95°C for 20 min with citrate antigen retrieval solution. These sections were incubated with primary antibodies Collagen-I (GB11022-3), Collagen-Ⅲ(GB111629), and *α*-SMA (GB111364) overnight, and then secondary antibodies were incubated with these sections for 50 min. Image-Pro Plus software (Media Cybernetics, United States) calculated cumulative optical densities.

### 2.9 Real-time quantitative PCR analysis

The mRNA expression levels of p53, p21, p16, and GAPDH in lung tissue were detected by rt-qPCR. Trizol reagent (Takara) extracted total RNA from lung tissue following the manufacturer’s protocol. Reverse transcription was carried out using the PrimeScript RT Reagent Kit (Takara). qRT-PCR was performed with the SYBR PrimeScript PCR kit II (Takara). A housekeeping gene, GAPDH, was used to standardize Ct values. Fold changes in mRNA expression were calculated by relative quantification (2^−ΔΔCt)^. Primer sequences used for PCR are shown in Table 2.

**TABLE 2:** Primer sequences used for rt-qPCR.

Gene name		Sequences
P53	Forward	ACA​GTT​AGG​GGG​TAC​CTG​GC
	Reverse	GAC​TCA​GAG​GGA​GCT​CGA​TG
P21	Forward	CCT​GGT​GAT​GTC​CGA​CCT​G
	Reverse	CCA​TGA​GCG​CAT​CGC​AAT​C
P16	Forward	GAG​GGC​TTC​CTA​GAC​ACT​CTG​GTA​G
	Reverse	AGA​TAC​CGC​AAA​TAC​CGC​ACG​AC
GAPDH	Forward	GAC​ATG​CCG​CCT​GGA​GAA​AC
	Reverse	AGC​CCA​GGA​TGC​CCT​TTA​GT

### 2.10 Western blotting analysis

Hub target proteins were subsequently validated by western blotting using specific antibodies. Briefly, we extracted proteins from rat lung tissue using lysates and protease inhibitors. Protein content was quantified with the BCA reagent kit. Proteins were separated by electrophoresis on SDS-PAGE gels and then transferred to the PVDF membranes. The membrane and primary antibodies were incubated overnight at 4°C after blocking with 5% skimmed milk. Following 5 washes with TBST, the membrane was incubated for 1 h with HRP-conjugated secondary antibodies. Finally, a chemiluminescence reagent was added to the membrane surface, and the imaging system visualized the target protein.

### 2.11 Enzyme-linked immunosorbent Assay

A quantitative ELISA was used to determine the levels of TNF-α, TGF-β1, and IL-6 in serum. The ELISA kits were all purchased from Jiubang Biotechnology (Fujian, China). All operations were performed strictly following the kit instructions. Briefly, serum samples (10 μl) and diluent solutions (40 ul) were separately added to the wells on a 96-well plate. Next, each well was added to HRP-labelled secondary antibodies and then incubated at 37°C for 60 min. Finally, we measured the optical density of each hole at 450 nm after adding 50 ml termination solution within 15 min.

### 2.12 Network pharmacology

#### 2.12.1 Obtaining the Yiqi Huayu decoction targets and PF-related gene sets

First, we screened the active ingredients of YHD from UHPLC-MS/MS analysis and Traditional Chinese Medicine Systems Pharmacology Database and Analysis Platform (TCMSP) (Ru et al., 2014). The screening criteria are that the oral bioavailability (OB) ≥ 30% and the drug-like (DL) index ≥0.18 (Li and Kan, 2021; Xia et al., 2020). Based on the active compound, we searched compound-related target genes in different databases (TCMSP, HERB, Swiss Target Prediction) (Gfeller et al., 2014; Ru et al., 2014; Fang et al., 2021). With the help of Uniprot, an AMSM target gene set is acquired after gene symbol annotation (Apweiler et al., 2004). Then, we searched PF-related genes in four databases: the Drugbank database, Genecards database, HERB database, and TTD database (Rebhan et al., 1997; Chen et al., 2002; Wishart et al., 2008; Fang et al., 2021). By combining the search results, we established a set of PF-related genes. PF-related genes and AMSM target genes were intersected to determine the common targets between drugs and diseases.

TCMSP database (https://www.tcmsp-e.com/).

Swiss Target Prediction web server (http://www.swisstargetprediction.ch).

HERB database (http://herb.ac.cn/).

Uniprot database (http://beta.uniprot.org/).

Drugbank database (https://go.drugbank.com).

Genecards database (https://www.genecards.org).

TTD database (http://db.idrblab.net/ttd/).

#### 2.12.2 Protein-protein interaction network and critical subnetwork

STRING database was used to construct the PPI network based on the common gene set (Damian et al., 2011). After setting the parameter as high confidence (0.9), the PPI network from STRING was imported into Cytoscape for further analysis. We applied two methods (CytoNca and CytoHubba) to screen the core subnetwork. Firstly, we used CytoNca(a plugin in Cytoscape) to analyze the PPI network (Tang et al., 2015). In detail, based on the primary score file calculated by CytoNca, we constructed a primary subnetwork consisting of the top 10 genes. Second, we used CytoHubba (another plugin in Cytoscape) to analyze the PPI network again (Chin et al., 2014). This approach analyzed the top 10 genes in the PPI network and constructed the critical subnetwork without checking the first-stage nodes.

STRING (https://www.string-db.org).

Cytoscape (version 3.8.2).

#### 2.12.3 Enrichment analysis and cluster analysis

A series of enrichment analyses were performed to determine the underlying mechanism, including gene ontology (GO), Kyoto Encyclopedia of Genes and Genomes (KEGG), and Reactome pathway analysis (Kanehisa and Goto, 2000; Harris et al., 2004; Joshi-Tope et al., 2005). KEGG and GO enrichment analysis was conducted using R’s “ClusterProfile” package (version 3.4.0) (Yu et al., 2012). The enrichment analysis of the Reactome pathway is completed using the DAVID database (Dennis et al., 2003). MCODE, a plugin for Cytoscape, carried out the clustering analysis. MCODE is mainly based on PPI network density and K-score for clustering analysis (The following parameters were set: degree cutoff = 2, node score cutoff = 0.2, K-score = 2, max. depth = 100) (Bader and Hogue, 2003).

### 2.13 Statistical analysis

The statistical analyses were conducted using GraphPad Prism version 8.0 (GraphPad Software, United States). All raw data are shown as mean ± SEM. A one-way ANOVA was performed after satisfying a normal distribution, followed by a Tukey multiple comparison test. *p*-values less than 0.05 were considered statistically significant.

## 3 Results

### 3.1 Components analysis of Yiqi HuaYu decoction

**FIGURE 2:**
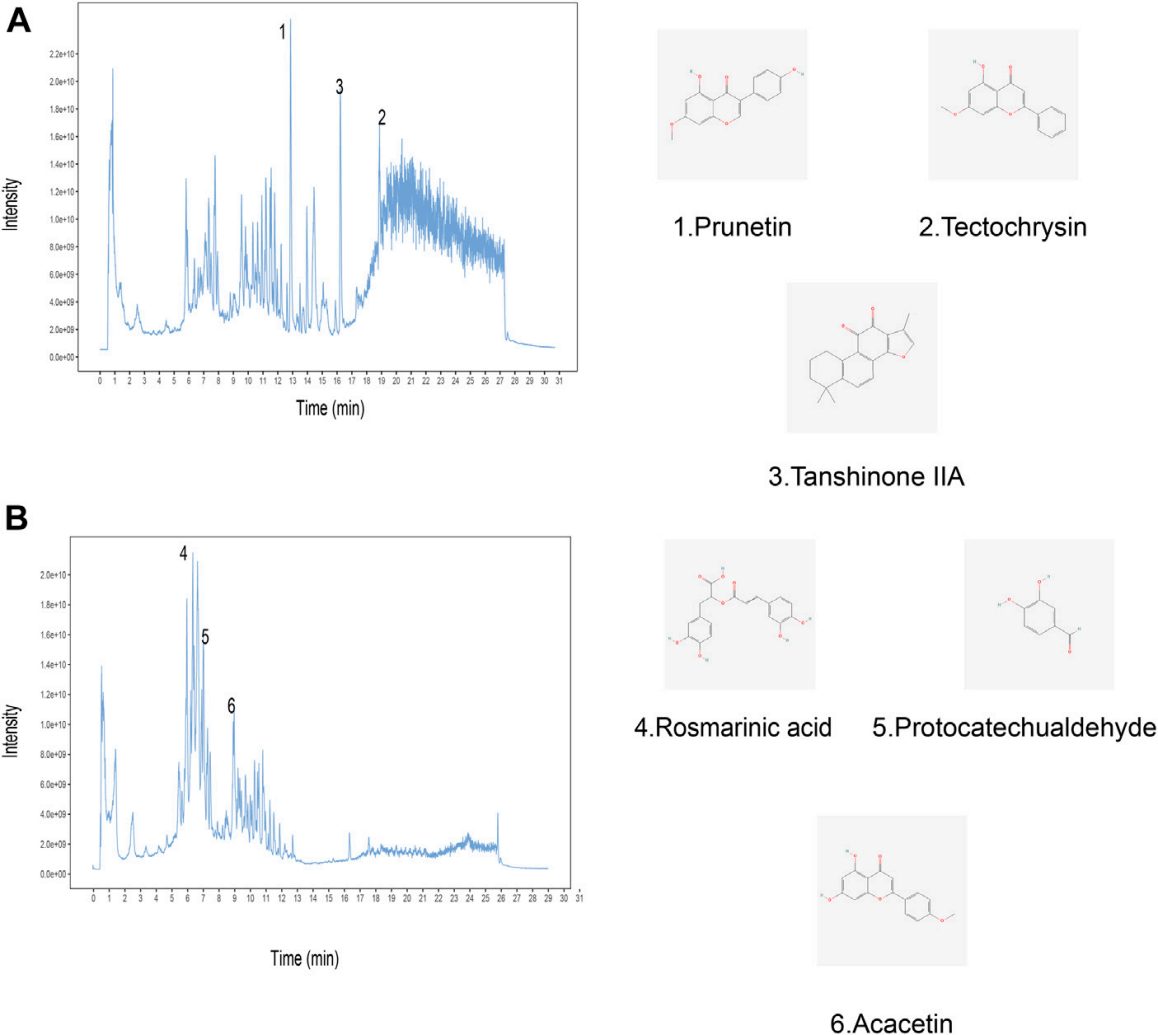
Identification of chemical components of YHD by ultra-high performance liquid chromatography-tandem mass spectrometry (UHPLC–MS/MS). Total ion chromatography in **(A)** positive and **(B)** negative ion modes for YHD samples is shown. The numbers correspond to the compounds on the left. The molecular structures of the compounds are on the right.

In order to identify the chemical components in Yiqi Huayu decoctions (YHD), UHPLC-MS/MS was used to analyze the samples. The total ion chromatograms (positive and negative) of YHD were obtained by UHPLC-MS/MS(Figures 2A–B). A total of 563 compounds were identified from the YHD samples. These compounds included 143 terpenoids, 86 flavonoids, 48 phenylpropanoids, 47 alkaloids, 45 miscellaneous, 34 phenols, 19 fatty acyls, 17 organic acids and their derivatives, 16 amino acid derivatives, 12 coumarins, *etc.* See the Supplementary Table S1 for detailed ingredient information. Figure 2 demonstrates six key compounds: 1) Prunetin, 2) Tectochrysin, 3) Tanshinone IIa, 4) Rosmarinic acid, 5) Protocatechualdehyde, and 6) Acacetin.

### 3.2 Yiqi HuaYu decoction alleviated bleomycin-induced pulmonary fibrosis in the rat

**FIGURE 3:**
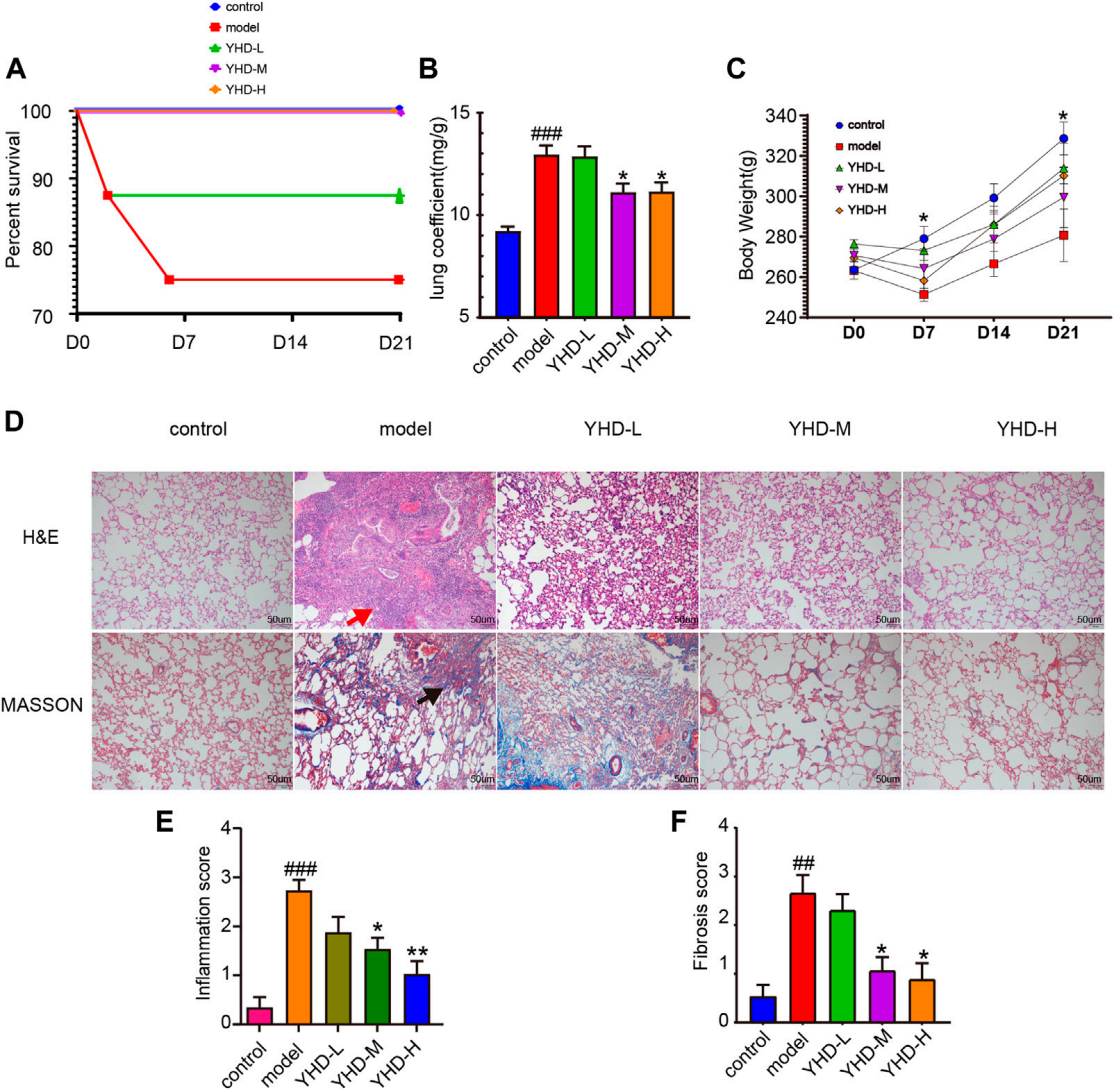
YHD alleviated BLM-induced PF in the rats. **(A)** Percent survival. Total two rats in the model group died (on day 2 and day 6, respectively), and one in the low-dose YHD group died (on day 2). No rats died in the remaining three groups. Each group of rats ended up with at least 6 biological replicates. **(B)**Lung coefficient. **(C)**Body weight. On day 7 and day 21, there was a statistical difference between the control and model group (n = 6). **(D)** Hematoxylin-Eosin and Masson staining (scale bar = 50 um). Thickening of the alveolar septum, destruction of alveolar structure, massive inflammatory cell infiltration in the alveolar space and interstitium (red arrows), and blue collagen fibers (black arrows). **(E–F)** Inflammation and fibrosis score. Inflammation and fibrosis scores were assessed based on HE-stained and MASSON-stained sections, respectively. Data were presented as the means ± SEM. ###*p* < 0.001, compared with the control group; *p < 0.05, **p < 0.01, compared with the model group, respectively.

Rat survival rates, body weights, and lung coefficients were recorded to verify the therapeutic effects of YHD on PF. Following intratracheal instillation of BLM at day 0, total two rats in the model group died (on day 2 and day 6, respectively), and one in the low-dose YHD group died (on day 2). Conversely, no rats died in the remaining three groups (Figure 3A). The lung coefficient can reflect the degree of inflammation and edema in the lung tissue (Shen et al., 2020). Rats in the model and YHD-L groups had a higher lung coefficient than those in the control group (*p* < 0.001). The medium- and high-dose YHD reduced the lung coefficients of fibrotic rats (Figure 3B). The body weight reflects the health status of fibrotic rats. Compared to the normal control group, the rats in the model group showed significant early weight loss (on day 7) and later slowed weight growth (on day 21). Different doses of YHD had a tendency to improve rat body weight, although there was no statistical difference (Figure 3C).

Pathological staining can more intuitively observe alveolar structure and degree of fibrosis. H&E staining showed obvious morphologic changes in the lung in the model group, including thickening of the alveolar septum, alveolar structure destruction, and heavy inflammatory cell infiltration in the alveolar space and lung interstitium (Figure 3D). In addition, the lung tissues of rats in the model group also displayed increased collagen deposition, as indicated by the increased blue fiber bundles in Masson-stained lung sections. The results of the inflammation and fibrosis scores showed that YHD treatment reduced the infiltration of inflammatory cells and decreased the deposition of collagen fibers. A significant dose effect was seen between the low-dose and high-dose groups (Figures 3E,F).

### 3.3 High-dose Yiqi HuaYu decoction improved lung function of fibrotic rats and inhibited collagen deposition in lung interstitium

**FIGURE 4:**
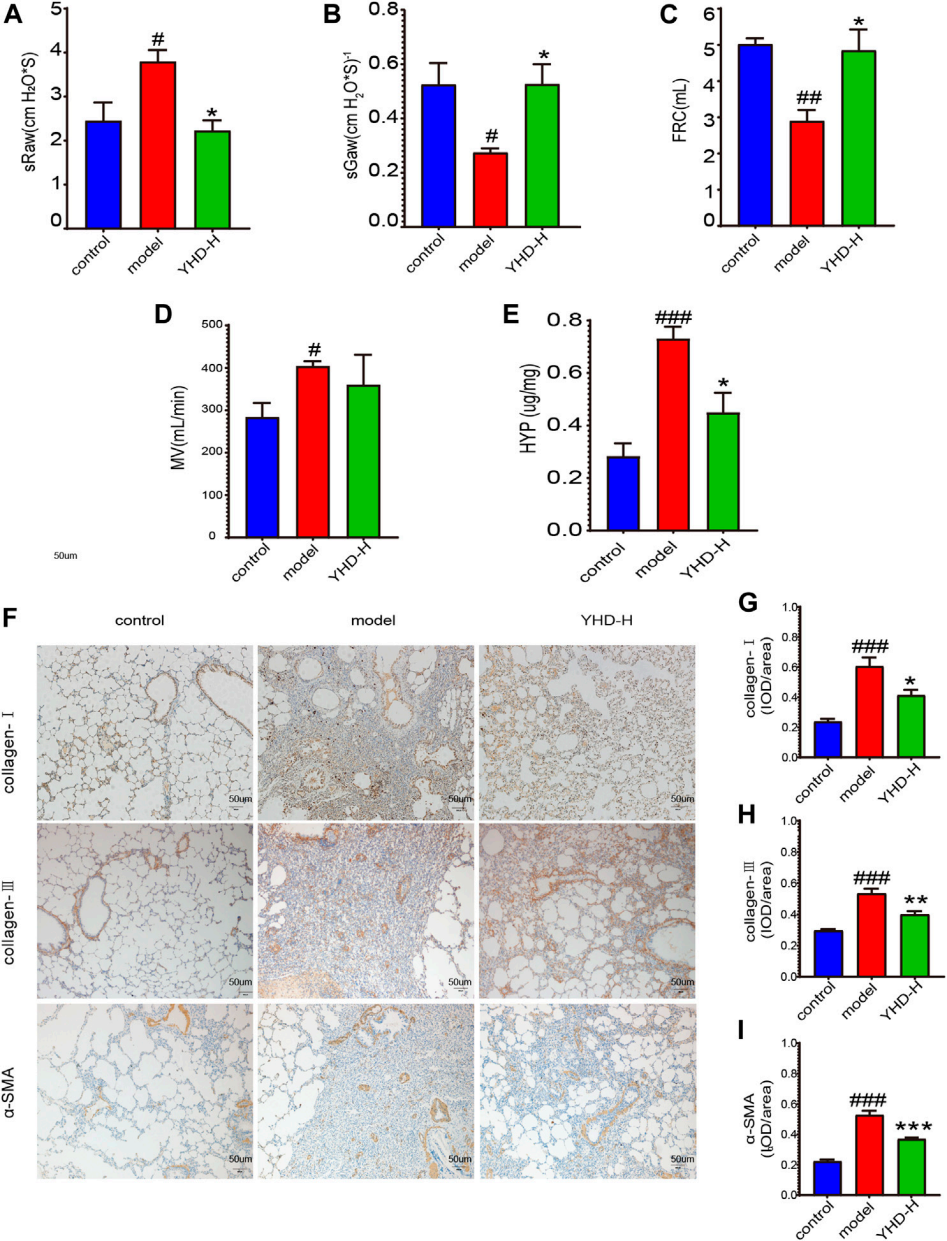
High-dose YHD improved lung function of fibrotic rats and inhibited collagen deposition in lung interstitium. **(A–D)** Lung function indicators: specific airway resistance (sRaw), specific airway conductance (sGaw), functional residual capacity (FRC), and minute volume (MV). **(E)** Hydroxyproline content. **(F)** Effects of high-dose YHD on pulmonary fibrosis by immunohistochemistry (scale bar = 50 um). **(G–I)** Image-Pro Plus software was used to statistically analyze the immunohistochemical staining results of collagen-Ⅰ, collagen-Ⅲ, and *α*-SMA. Data were presented as the means ± SEM (*n* = 6). #*p* < 0.05, ##*p* < 0.01, ###*p* < 0.001, compared with the control group; *p < 0.05, **p < 0.01, ***p < 0.01, compared with the model group, respectively.

It is well known that PF can compromise lung function. Since high-dose YHD exhibited better therapeutic effects, we evaluated the improvement effect of high-dose YHD on lung function. High airway resistance is indicated by increased specific airway resistance (sRaw) and decreased specific airway conductance (sGaw). Decreased functional residual capacity (FRC) suggests alveolar contraction or collapse. Lung function results showed that sRaw and minute volume (MV) were elevated, while sGaw and FRC were decreased in model rats compared to control rats. After using high-dose YHD, sGaw and FRC increased, and sRaw decreased compared with the model rat (Figures 4A–D). These data indicated that high-dose YHD alleviated BLM-induced alveolar collapse and decreased airway resistance. High expression of *α*-SMA and excessive collagen deposition (mainly type I and type III) are characteristics of PF. By immunohistochemical staining of lung sections, we observed that a large amount of collagen (type I and type III) and *α*-SMA proteins were deposited in the lung interstitium of rats in the model group. However, high-dose YHD significantly reduced the expression of collagen protein (type Ⅰ and Ⅲ) and *α*-SMA (Figures 4F–I). In addition, hydroxyproline (HYP), an essential component of collagen synthesis, was also measured (Medugorac, 1980). The lung tissue of rats in the model group contained more HYP compared with the control group (*p* < 0.001). Conversely, high-dose YHD decreased lung hydroxyproline levels in fibrotic rats (Figure 4E). These results indicate that YHD can reduce collagen deposition and activation of myofibroblasts.

### 3.4 Network pharmacology

#### 3.4.1 Identification of the theoretical active compounds and proteins targets of Yiqi HuaYu decoction

**FIGURE 5:**
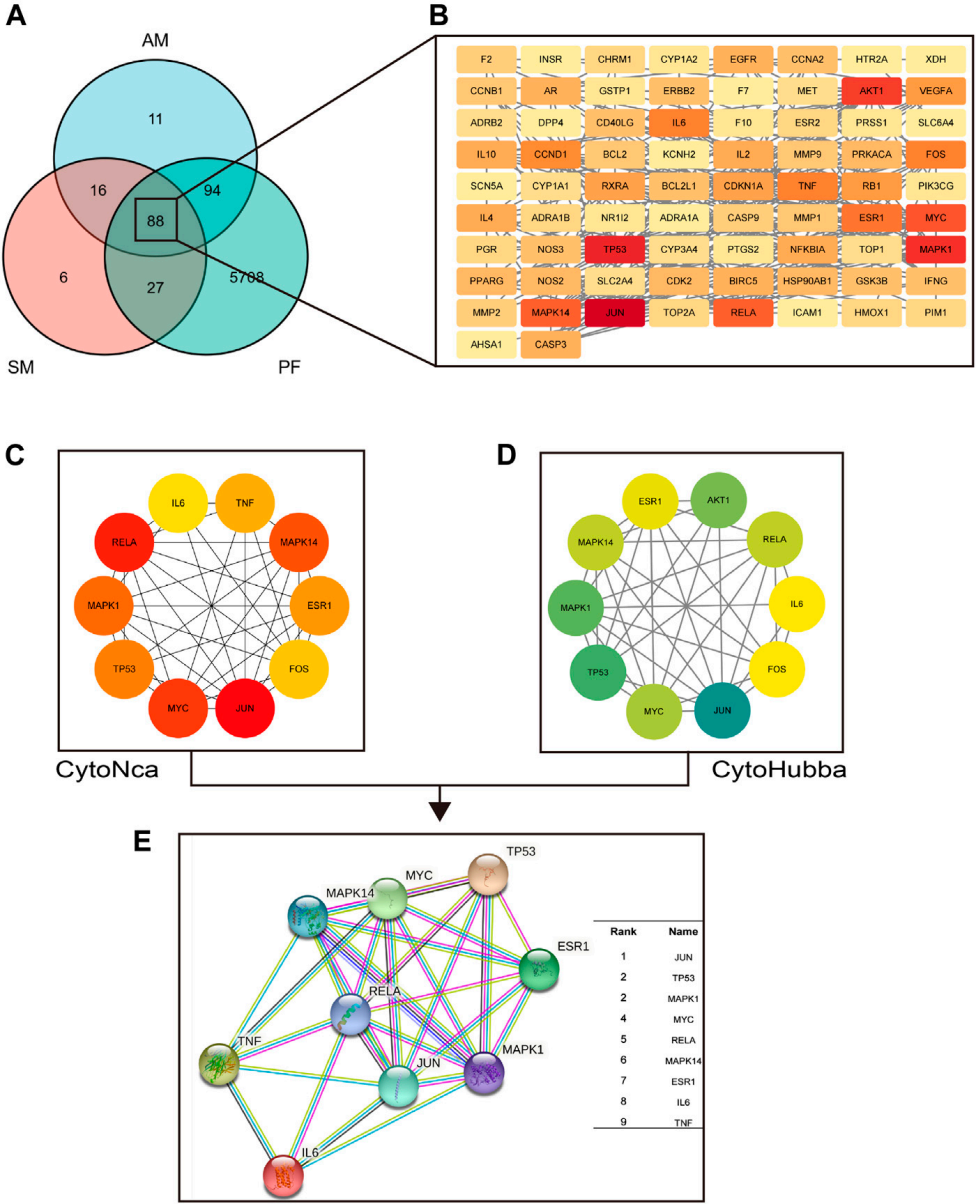
The hub target proteins of YHD in treating PF screened by network pharmacology. **(A–B)** the common target proteins between YHD and PF. Red nodes represent critical target proteins. **(C)** the top 10 target proteins screened by CytoNca algorithms. **(D)** the top 10 target proteins screened by CytoHubba algorithms. **(E)** the nine most critical target proteins obtained from the intersection. Two different algorithms screened target proteins, and the intersection was taken to obtain the most critical target proteins.

YHD effective compounds were collected from UHPLC-MS/MS analysis and the TCMSP database. Compounds that meet the requirements of oral bioavailability (OB) ≥ 30% and drug-like (DL) index≥0.18 can be considered potential drug candidate active ingredients (Xia et al., 2020). We screened 20 active compounds of *Astragalus* menbrunucrus (AM) and 65 active compounds of Salvia miltiorrhiza (SM). Supplementary Table S2 contains detailed information on these active compounds. Then, using the screened active compounds, we identified 209 a.m. target genes and 137 S M target genes from databases (TCMSP, HERB, and Swiss Target Prediction). In addition, we identified 5,917 pulmonary fibrosis (PF) target genes from databases (Drugbank, Genecards, HERB, and TTD). Finally, by taking the intersection, we get 88 overlapping common protein targets among AM, SM, and PF (Figure 5A). These common targets were used for further analysis. Supplementary Table S3 includes all target detail information.

#### 3.4.2 Hub target proteins and critical signal pathway

We could identify hub target proteins through the protein-protein interaction (PPI) network. Figure 5B shows the PPI network obtain from the STRING database. The nodes in red represent key hub proteins. In addition, we used two algorithms (CytoHubba and CytoNCA) to find the critical top ten proteins in 88 common targets (Figures 5C,D). Finally, by taking the intersection, we found nine key proteins (Figure 5E): JUN, TP53, MAPK1, MYC, RELA, MAPK14, ESR1, IL-6, and TNF.

**FIGURE 6:**
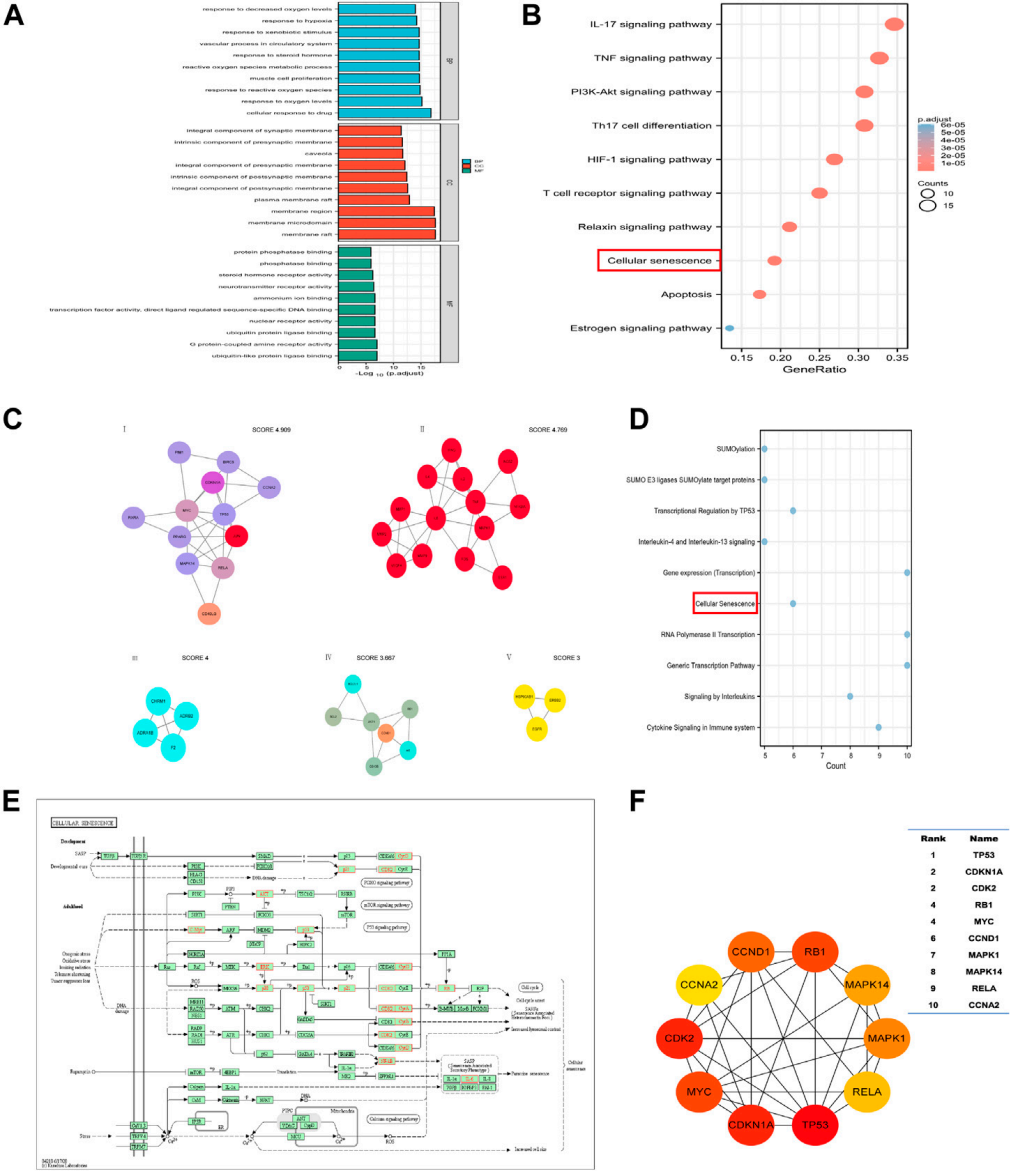
The critical pathways screened by network pharmacology. **(A)** GO enrichment analysis. **(B)** KEGG enrichment analysis. Cellular senescence signaling pathways were enriched for the first time. **(C)** The five clusters screed by MCODE analysis. **(D)** Reactome pathway enrichment analysis of the cluster Ⅰ. The cellular senescence signaling pathway was again enriched. **(E–F)** The cellular senescence pathway and key markers (TP53/P53, CDKN1A/P21) on the pathway.

Enrichment analysis can identify key signaling pathways and biological functions. The categories covered by GO enrichment analysis were molecular function (MF), cellular component (CC), and biological process (BP). Based on the 88 common targets, we obtained 174 KEGG pathways and 2513 GO terms, including 2266 BP, 81 CC, and 166 MF. As shown in Figure 6A, the top 10 terms in BP, CC, and MF are listed. The most enriched BP terms were associated with oxygen metabolism, including response to oxygen levels, response to reactive oxygen species, reactive oxygen species metabolic process, response to hypoxia, and response to decreased oxygen levels. We screened out the top 10 signaling pathways related to PF (Figure 6B). These pathways are mainly related to the inflammatory response (IL-17 signaling pathway and TNF signaling pathway), immune response (Th17 cell differentiation and T cell receptor signaling pathway), and cell cycle regulation (apoptosis and cellular senescence).

Molecular Complex Detection (MCODE) analysis can screen out key functional modules of molecular networks. Through MCODE, we obtained 5 key cluster subnetworks (Figure 6C). Each clustering subnetwork is scored, with higher scores representing more critical. We performed Reactome pathway analysis on the first cluster subnetwork and found that cellular senescence signaling pathways were enriched again (Figure 6D). Figure 6E shows a cellular senescence signaling pathway diagram, with red labels representing 13 target proteins enriched in this pathway. Figure 6F shows the top ten proteins among the 13 senescence-related proteins, and the upper right table shows their degree rankings.

### 3.5 High-dose Yiqi HuaYu decoction inhibited cellular senescence

**FIGURE 7:**
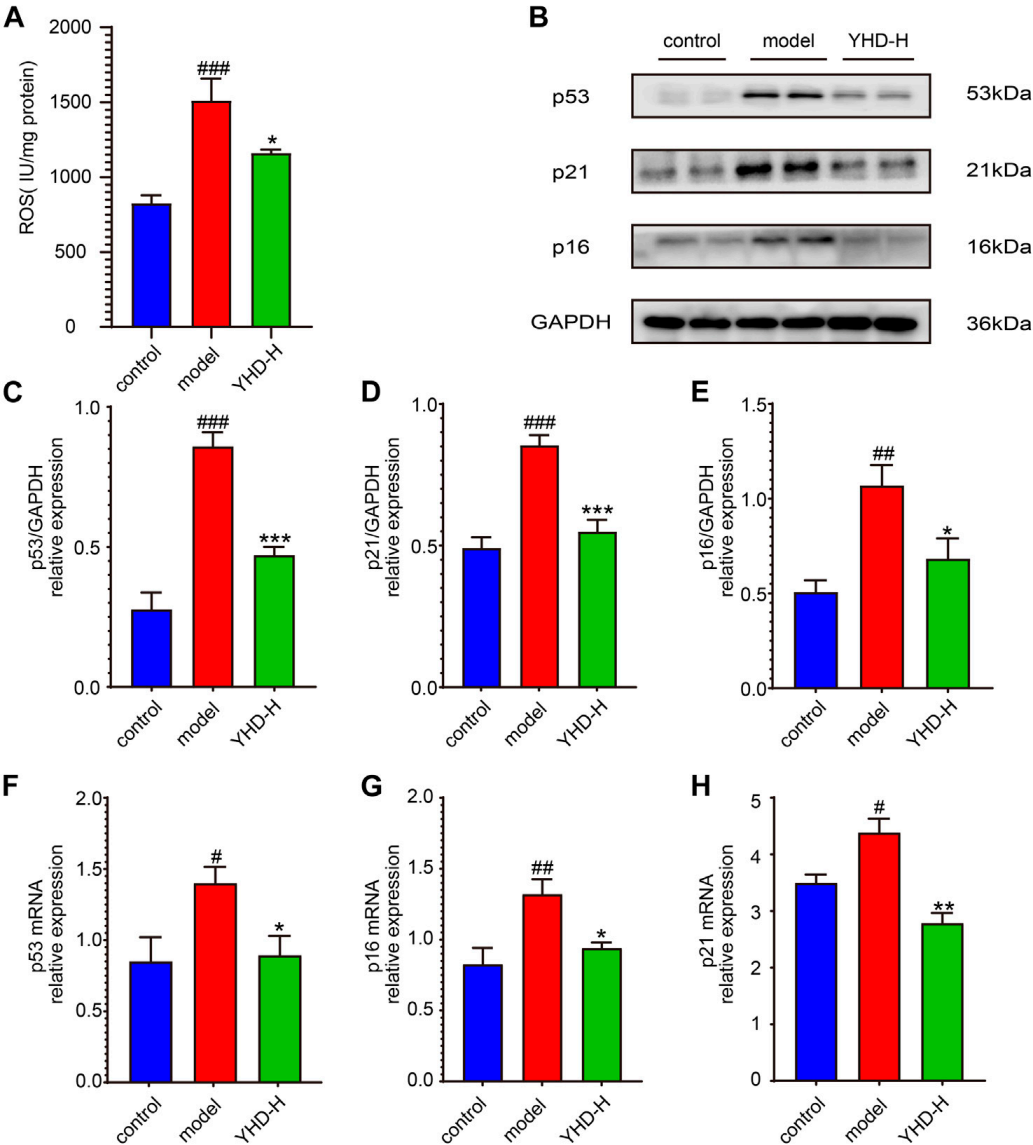
High-dose YHD inhibited cellular senescence. **(A)** the relative content of ROS in the lung tissue. **(B)** Western blotting of p53, p21, and p16 in the lung of fibrotic rats. **(C–E)** The relative protein expression of p53, p21, and p16 in the lung. **(F–H)** The relative gene expression of p53, p21, and p16 in the lung by rt-qPCR. Data were presented as the means ± SEM (*n* = 6 each group). #*p* < 0.05, ##*p* < 0.01, ###*p* < 0.001, compared with the control group; *p < 0.05, **p < 0.01, ***p < 0.001, compared with the model group.

Because the cellular senescence pathway is enriched multiple times, we examined the key markers TP53 (p53) and CDKN1A (p21) on this pathway. In addition, CDKN2A (p16), another key marker of cellular senescence, was detected despite not being captured by network pharmacology. On both the protein and gene levels, the lung tissues of the model rats showed significantly higher expression of p53, p21, and p16 than the control group. Nevertheless, high-dose YHD significantly decreased the expression of these senescence markers (Figures 7B–H).

Increased reactive oxygen species (ROS) can induce cellular senescence, so we examined ROS expression in lung tissue (Figure 7A). As expected, the model rats had significantly higher levels of ROS in their lung tissue than the control rats (*p* < 0.001), while high-dose YHD could reduce its expression (*p* < 0.05).

**FIGURE 8:**
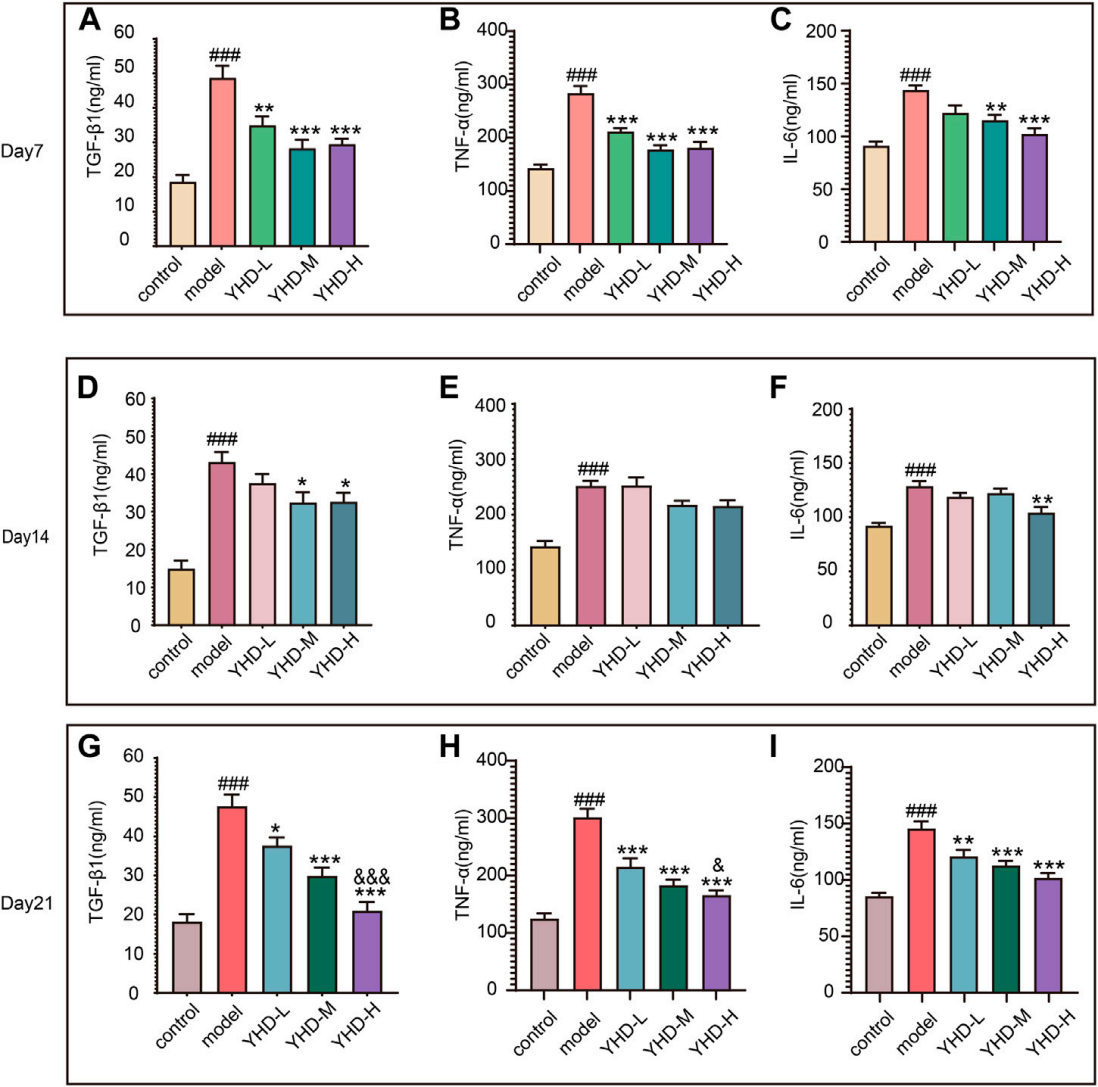
High-dose YHD inhibited SASPs. Senescent cells can secrete a combination of factors collectively known as the SASPs, including TGF-β1, TNF-α, and IL-6. The content of TGF-β1, TNF-α, and IL-6 in rat orbital blood by ELISAs once a week (on day 7, day 14, and day 21). Data were presented as the means ± SEM (*n* = 6 each group). #*p* < 0.05, ##*p* < 0.01, ###*p* < 0.001, compared with the control group; *p < 0.05, **p < 0.01, ***p < 0.001, compared with the model group; &*p* < 0.05, &&*p* < 0.01, &&&*p* < 0.001, compared with the low-dose YHD group.

Senescent cells can secrete various cytokines collectively known as the SASPs, including TGF-β1, TNF-α, and IL-6. SASPs can reinforce the senescence program and influence the tissue microenvironment. Therefore, we detected the contents of SASPs (TGF-β1, TNF-α, and IL-6) in rat orbital blood by ELISA once a week. ELISA results showed that the levels of TGF-β1, TNF-α, and IL-6 in the serum of model rats were significantly increased on day 7, 14, and 21 compared with the control group (*p* < 0.001). Conversely, different doses of YHD could decrease the content of these SASPs and show a significant gradient effect (Figures 8A–I).

Taken together, these results suggest that YHD can inhibit senescence and SASPs.

## 4 Discussion

**FIGURE 9:**
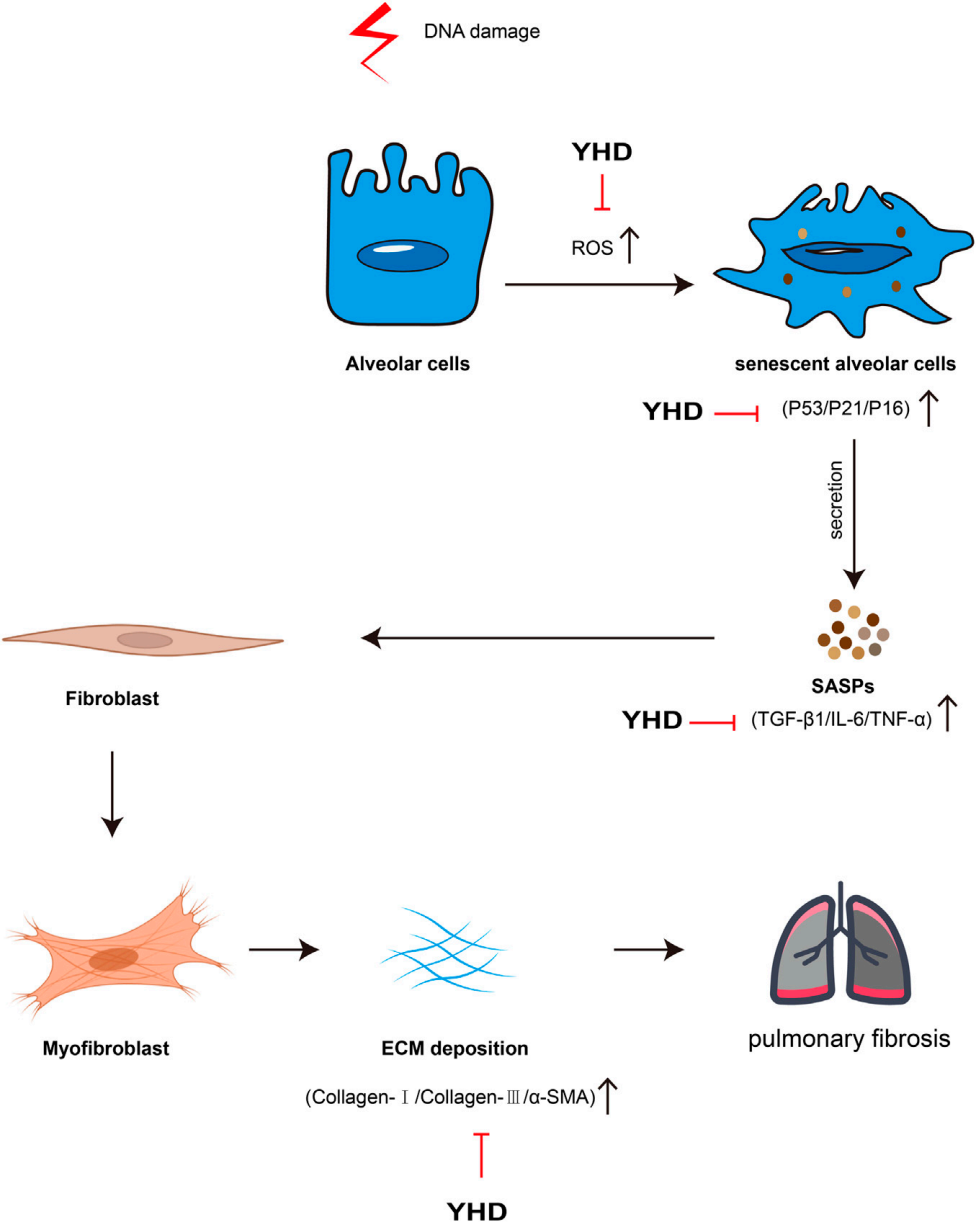
Schematic of Mechanism of YHD in treating PF. The red arrows indicate inhibitory effects.

There is currently no cure for PF, which frequently leads to organ failure and death (Moss et al., 2022). TCM is an excellent resource for drug innovation discovery, and we try to find shortcuts to treating PF from this treasure trove. Practitioners of TCM believe that the pathogenesis of PF is mainly Qi deficiency and blood stasis, so the therapy strategy is to tonify qi, activate blood, and remove stasis (Li and Kan, 2017). YHD consists of two botanical drugs: *Astragalus* mongholicus Bunge (AM) and Salvia miltiorrhiza Bunge (SM). Data mining analysis of TCM clinical prescriptions showed that AM and SM were the most frequently used herbs for tonifying Qi and activating Blood, respectively (Zhang et al., 2018). In our study, we confirmed the efficacy of YHD in an animal model and preliminarily explored the underlying mechanisms of this drug. The mechanism of YHD for PF is shown in Figure 9. Our study lays the foundation for developing novel drugs for PF while providing evidence support for the clinical promotion of YHD.

The diagnosis of PF and assessment of drug efficacy are difficult in clinical practice (Richeldi et al., 2017). Therefore, we assessed the efficacy of YHD by a comprehensive evaluation of body weight, lung coefficients, lung function, and pathological lung structures. We used BLM for the PF model, which is currently the most widely used animal model (Martinez et al., 2017). Our study demonstrated that YHD treatment significantly improved BLM-induced weight loss and pathological lung changes in rats. Excessive collagen deposition and myofibroblast activation are features of PF, and the high expression of *α*-SMA is a marker of myofibroblast activation (Moss et al., 2022). Previous studies have shown that YHD can inhibit collagen deposition (He et al., 2012). Consistent with their results, we found that YHD notably decreased the content of collagen (type Ⅰ and type Ⅲ) and *α*-SMA in fibrotic lung tissues. To better meet clinical criteria, we examined fibrotic rats’ lung function. YHD could significantly improve high airway resistance in fibrotic rats. To summarize, these results demonstrated that YHD mitigated BLM-induced PF.

Cellular senescence is thought to be a major driver of the malignant progression of PF (Schafer et al., 2017). The process of cellular senescence is caused by multiple physiological and pathological stresses that result in a permanent cell cycle arrest (Muñoz-Espín and Serrano, 2014). Cellular senescence consists of replicative and stress-related senescence (Hernandez-Segura et al., 2018). Replicative senescence is mainly due to the activation of p53 after DNA damage. Stress-related senescence is mainly due to oxidative stress damage, which activates the cyclin-dependent kinase inhibitor p16. Both pathways can activate the downstream cyclin-dependent kinase inhibitor p21 (Barnes et al., 2019). Activating p21 causes cells to exit the cell cycle, resulting in a permanent cell cycle arrest (Lv et al., 2021). In a recent study, researchers found that p53 and p21 were significantly highly expressed in the lung tissue of PF patients. Moreover, pifithrin-α, a specific p53 inhibitor, can reduce the senescence of type II alveolar cells, thereby alleviating experimental pulmonary fibrosis (Yao et al., 2021). We found that YHD inhibited cellular senescence markers, as evidenced by decreased gene and protein expression of p53, p21, and p16.

Despite being in a state of cell cycle arrest, senescent lung epithelial cells are still metabolically active (Yao et al., 2021). These senescent cells can secrete various growth factors, cytokines, and proteases, known as SASPs. Unfortunately, the SASPs cause low-grade chronic inflammation, which further triggers senescence and massive accumulation of extracellular matrix (Hernandez-Segura et al., 2018). Our experiment dynamically detected the levels of SASPs (TGF-β1, TNF-α, and IL-6) in rat serum. ELISA results showed that the progression of PF was accompanied by continuous stimulation of these SASPs. High-dose YHD consistently suppressed the expression of these SASPs in serum during the progress of PF. These results suggest that YHD can inhibit cellular senescence and SASPs, which may be beneficial in explaining the anti-fibrotic mechanism of YHD.

However, our study has some limitations. Because YHD intervention is difficult to perform on cell experiments, next, we will continue to screen active compounds of YHD and further explore their anti-fibrotic mechanisms.

## 5 Conclusion

In conclusion, our study suggests that YHD improves lung pathological injury and lung function in PF rats. This protective effect may be related to the ability of YHD to inhibit cellular senescence.

## Data Availability

The datasets presented in this study can be found in online repositories. The names of the repository/repositories and accession number(s) can be found in the article/Supplementary Material.

## Abbreviations

α-SMA, α-smooth muscle actin; AM, *Astragalus* membranaceus (Fisch.) Bunge; BLM, bleomycin; ELISAs, enzyme-linked immunosorbent assays.; FRC, functional residual capacity; GO, gene ontology; H&E, Hematoxylin-Eosin stain; HYP, hydroxyproline; IL-6, interleukin 6; KEGG, Kyoto encyclopedia of genes and genomes; MCODE, molecular complex detection analysis; PF, pulmonary fibrosis; PPI, protein-protein interaction; ROS, reactive oxygen species; Rt-qPCR, real-time quantitative polymerase chain reaction; SASPs, senescence-associated secretory phenotypes; sGaw, specific airway conductance; SM, Salvia miltiorrhiza Bunge; sRaw, specific airway resistance; TCM, traditional Chinese medicine; TGF-β1, transforming growth factor-β1; TNF-α, tumor necrosis factor-alpha; UHPLC-MS/MS, ultra-high-performance liquid chromatography tandem mass spectrometry; WB, western blot; YHD, yiqi huayu decoction.
