# Supplementary material for: Yiqi Huayu decoction alleviates bleomycin-induced pulmonary fibrosis in rats by inhibiting senescence
Source: Front Pharmacol. 2022 Oct 28;13:1033919. doi: 10.3389/fphar.2022.1033919 (PMC9649452; doi:10.3389/fphar.2022.1033919)
Supplement: Supplementary file 1 [file DataSheet1.ZIP › Supplementary material Inflammation and fibrosis scoring criteria.docx]

**Alveolitis was evaluated with the hemotoxylin and eosin-stained sections and was graded using the following criteria:**

none (0): no alveolitis;

mild (1+): thickening of the alveolar septum by a mononuclear cell infiltrate, with involvement limited to focal, pleural-based lesions occupying less than 20 % of the lung and with good preservation of the alveolar architecture;

moderate (2+): more widespread alveolitis involving 20 to 50 % of the lung, although still predominantly pleural based;

severe (3+): diffuse alveolitis involving more than 50 % of the lung, with occasional consolidation of air spaces by the intra-alveolar mononuclear cells and some hemorrhagic areas within the interstitium and /or alveolus.

**Fibrosis was evaluated using Masson's trichrome stain sections. The extent of fibrosis in these sections was graded using the following criteria:**

none (0): no evidence of fibrosis;

mild (1+): focal regions of fibrosis involving less than 20 % of the lung. Fibrosis involved the pleura and the interstitium of the subpleural parenchyma with some distortion of alveolar architecture;

moderate (2+): more extensive fibrosis involving 20 to 50 % of the lung and fibrotic regions mostly extending inward from the pleura and still focal;

severe (3+): widespread fibrosis, involving more than 50 % of the lung. Confluent lesions with extensive derangement of parenchymal architecture, including cystic air spaces lined by cuboidal epithelium.

**Detailed evaluation methods refer to this classic literature:**

(1). Szapiel, S. V., Elson, N. A., Fulmer, J. D., Hunninghake, G. W., & Crystal, R. G. (1979). Bleomycin-induced interstitial pulmonary disease in the nude, athymic mouse. American Review of Respiratory Disease, 120(4), 893-899.
